# Bis(μ-benzoato-κ^2^ *O*:*O*′)bis­(benzoato-κ*O*)octabutyldi-μ_3_-oxido-tetra­tin(IV)

**DOI:** 10.1107/S2056989017001505

**Published:** 2017-02-03

**Authors:** Hans Reuter, Coco K. Y. A. Okio

**Affiliations:** a Institute of Chemistry of New Materials, University of Osnabrück, Barbarastrasse 7, 49069 Osnabrück, Germany; b Departamento de Química, Facultad de Ciencias, Universidad Nacional de Colombia, Carerra 30 No 45-03, Bogotá, Colombia

**Keywords:** crystal structure, di­carboxyl­ate, tetra­organodistannoxane, ladder structure

## Abstract

The asymmetric unit of the title compound, [{Sn(C_4_H_9_)_2_(C_6_H_5_COO)}_2_O]_2_, consists of two half molecules, completed by application of inversion symmetry. Both mol­ecules adopt a ladder structure typical for this class of dimeric tetra­organodistannoxane di­carboxyl­ates characterized by a centrosymmetric four-membered (Sn—O)_2_ ring of rhomboidal shape that is extended on both sides by folded six-membered Sn—O—C rings. To a first approximation, both kinds of Sn atoms (Sn_*i*_ and Sn_*o*_) are trigonal–bipyramidally coordinated. The bond angles between the *n*-butyl groups are widened [135.64 (7)–146.20 (7)°] in comparison with an ideal trigonal bipyramid. Sn—O bond lengths within the {*R*_2_SnO_3_} coordination sphere depend strongly on the position of the corresponding O atom – axial (*ax*) or equatorial (*eq*) – as well as on the functionality of the carboxyl­ate groups which exhibit μ_2_ (–COO_*i*_) and μ_1_ (–COO_*o*_) coordination modes, respectively. In summary, the following sequence of distances [mean values] is found: *d*(Sn_*o*_—Oμ_3_)_*eq*_ [2.024 (2) Å] < *d*(Sn_*i*_—Oμ_3_)_*eq*_ [2.044 (2) Å] < *d*(Sn_*i*_—Oμ_3_)_*ax*_ [2.158 (6) Å] < *d*(Sn_*o*_—Oμ_1_-carb)_*ax*_ [2.182 (6) Å] < *d*(Sn_*i*_—Oμ_2_-carb)_*ax*_ [2.250 (2) Å] ≃ *d*(Sn_*o*_—O*μ_2_-carb*)_*ax*_ [2.247 (12) Å]. The *n*-butyl groups adopt an *anti–anti* conformation with exception of two disordered outer *n*-butyl groups of the second mol­ecule which exhibit *gauche–anti* and *anti–gauche* conformations. Weak intra­molecular Sn⋯O inter­actions between the different O atoms of the outer carboxyl groups with the inner, as well as outer, Sn atoms give rise to a strongly distorted octa­hedral coordination at these Sn atoms. Inter­molecular inter­actions between the individual mol­ecules are restricted to van der Waals and O⋯H—C inter­actions of which a nearly linear very short C—H⋯O contact between the H atom of the phenyl group of one of the mol­ecules with the outer non-coordinating C=O group of the other molecule is the most prominent. It gives rise to a chain-like arrangement of the mol­ecules along [111]. The two *n*-butyl groups attached to the outer Sn atom of one mol­ecule are disordered over two sets of sites with occupancies of 0.806 (3)/0.194 (3) and 0.702 (3)/0.298 (3).

## Abstract 2

The asymmetric unit of [{Sn(C_4_H_9_)_2_(C_6_H_5_COO)}_2_O]_2_ consists of two independent centrosymmetric half formula units. Both mol­ecules adopt the ladder structure typical for this class of dimeric tetra­organodistannoxane di­carboxyl­ates. A nearly linear very short C—H⋯O contact gives rise to a chain-like arrangement of mol­ecules in the [111] direction

## Chemical context   

Our focus on organotin(IV) carboxyl­ates is due to the variety of architectures and the diverse applications displayed by those compounds (Davies, 1997 ▸; Chandrasekhar *et al.*, 2008 ▸). In our search of new structures displayed by these compounds and their derivatives, we recently reported the structure of monomeric di-*n*-butyl­tin (IV) dibenzoate, *n*Bu_2_Sn(OOCPh)_2_ (Reuter & Okio, 2016 ▸), with the tin atom sixfold coordinated *via* intra­molecular complexation. While that compound has been synthesized by the reaction of di-*n*-butyl­tin(IV) oxide, *n*Bu_2_SnO, with benzoic acid, PhCOOH, in a molar ratio of 1:2, we herein present the structure of [{*n*Bu_2_Sn(OOCPh)}_2_O]_2_ obtained from the same reactants using a molar ratio of 1:1.
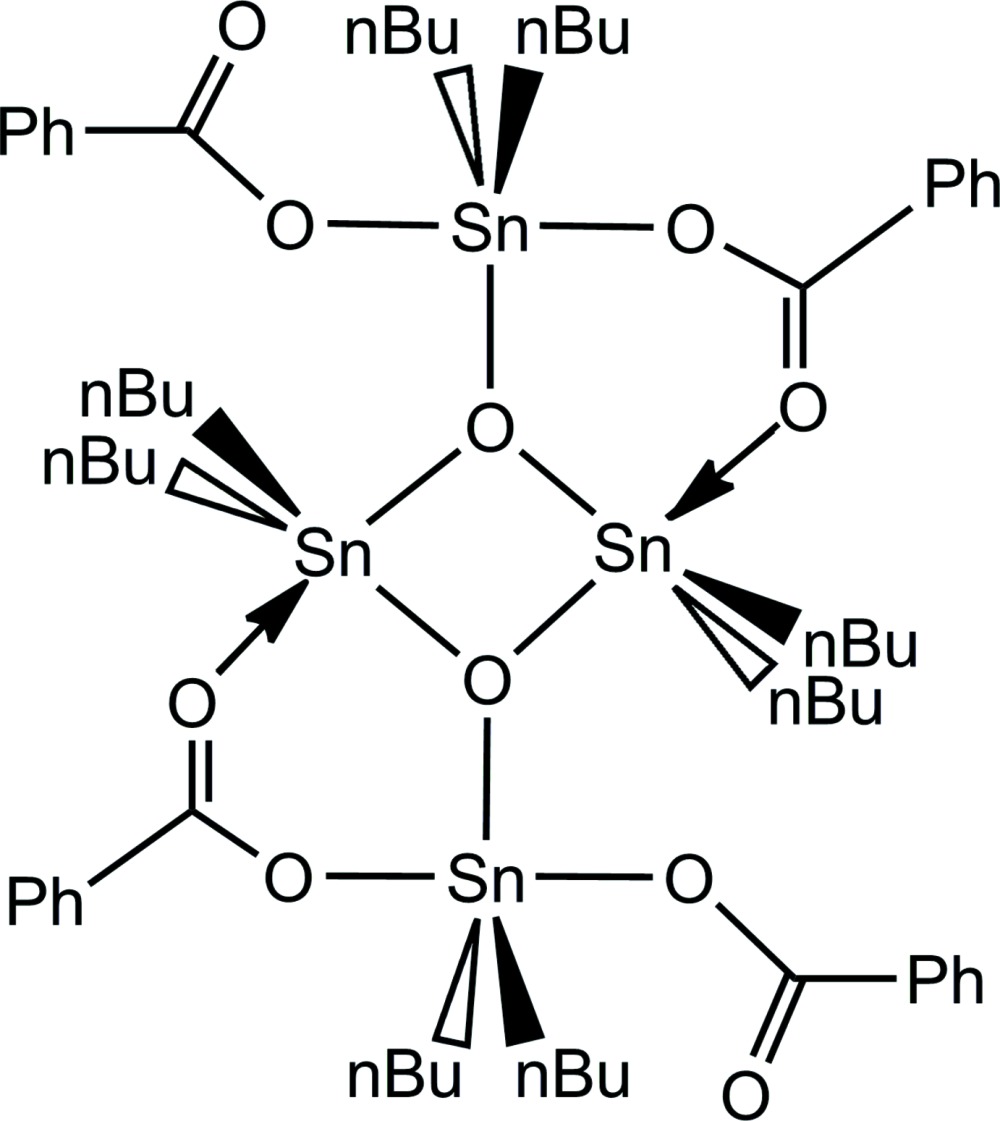


## Structural commentary   

**Figure 1:**
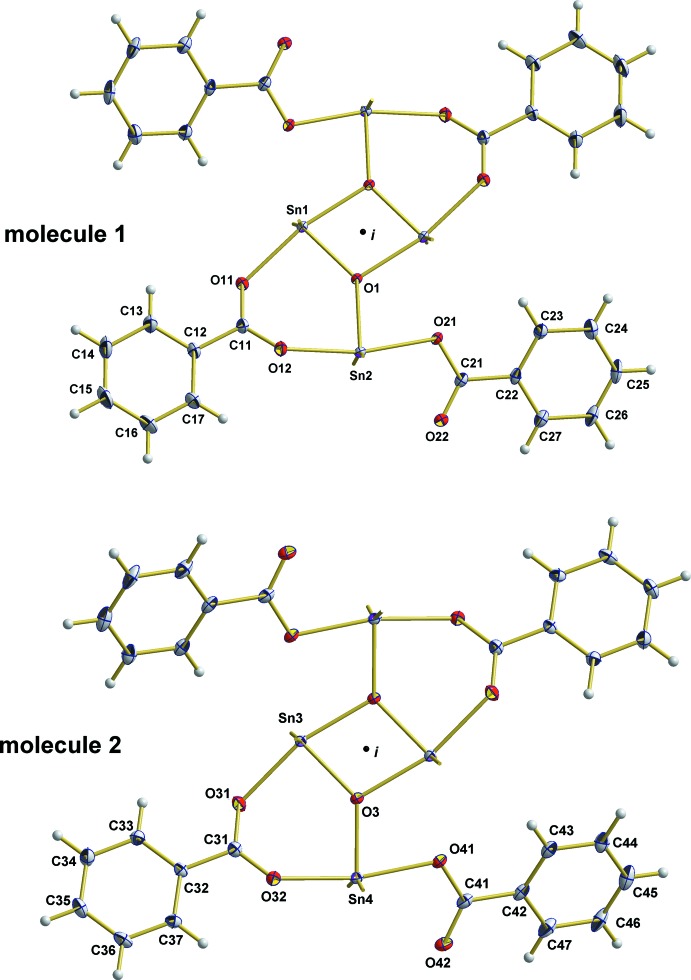
The asymmetric unit (labelled atoms) of the title compound, showing the atom-labeling scheme and displacement ellipsoids of the non-H atoms at the 50% probability level. *n*-Butyl groups have been omitted for clarity.

**Figure 2:**
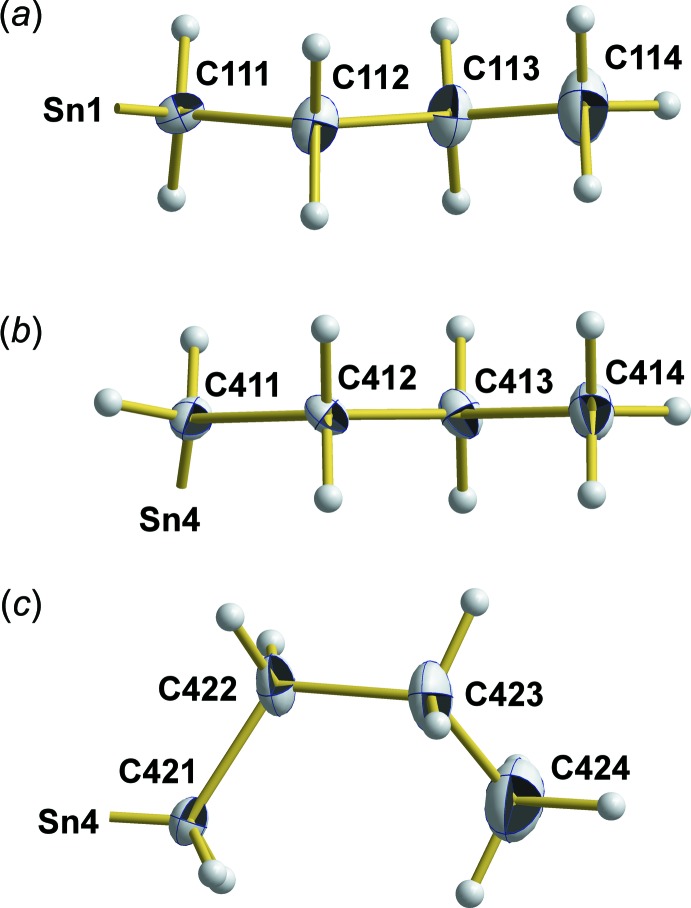
Main types of conformations adopted by the *n*-butyl groups of the title compound: (*a*) *anti–anti*, (*b*) *anti–gauche* and (*c*) *gauche–anti*. Displacement ellipsoids of the non-H atoms are drawn at the 50% probability level and bonds to Sn atoms are indicated as short sticks.

The title compound (Fig. 1 ▸) crystallizes with two formula units [{*n*Bu_2_Sn(OOCPh)}_2_O]_2_ in space group *P* 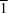. The asymmetric unit consists of one formula unit composed of two half molecules, completed by application of inversion symmetry (Fig. 1 ▸). With the exception of both *n*-butyl groups attached to the outer Sn atom (Sn4) of the second mol­ecule, all parts of the structure are well ordered. The disorder of the two *n*-butyl groups was managed by a split model with site occupancies of 0.806 (3)/0.194 (3) and 0.702 (3)/0.298 (3). No further consideration will be made for the structural parameters of those *n*-butyl groups. It is noteworthy, however, that this disorder is caused by the conformational flexibility of the *n*-butyl group which adopts – in the case of the major/minor components – a *gauche*–*anti*/*anti–gauche* and *anti*-*gauche*/*anti–gauche* conformation with respect to the Sn—C_α_ **—**C_β_—C_γ_ and C_α_—C_β_ **—**C_γ_—C_δ_ torsion angles (Fig. 2 ▸ *b*,*c*). This conformation of the disordered *n*-butyl groups is in contrast to the conformation of all other *n*-butyl groups of both dimers, which show exclusively an *anti*–*anti* conformation (Fig. 2 ▸ *a*). Structural parameters (Table 1 ▸) within the ordered *n*-butyl groups follow the general trends: *d*(C—C)_mean_ = 1.521 (6) Å, 〈(C—C_β/γ_—C)_mean_ = 112.6 (11)° while bond angles at C_α_ range from 112.5 (1) to 123.1 (1)°. Sn—C distances are in the range of 2.127 (2)–2.134 (2) Å, mean value 2.130 (3) Å.

**Figure 3:**
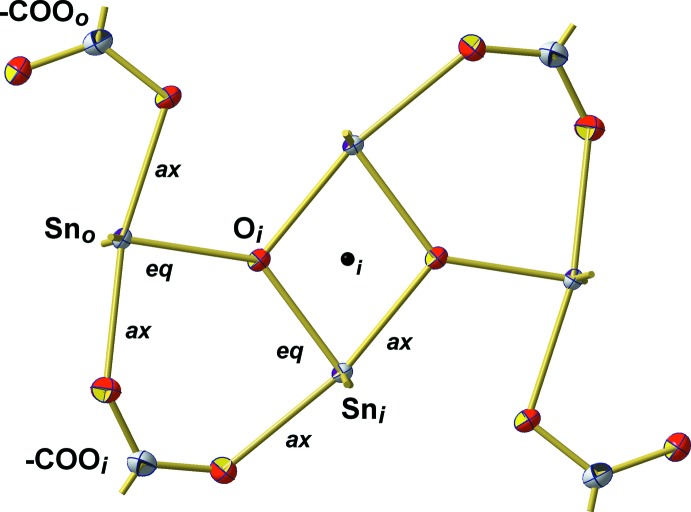
Location of axial (*ax*) and equatorial (*eq*) bonds with respect to the trigonal–bipyramidal coordination at the inner (Sn_*i*_) and outer (Sn_*o*_) Sn atoms and of the outer (–COO_*o*_) μ_1_- and inner (–COO_*i*_) μ_2_-carboxyl­ate groups.

The Sn–O framework of both mol­ecules adopts the *ladder* structure typical for this class of tetra­organodistannoxane di­carboxyl­ates (Chandrasekhar *et al.*, 2008 ▸). This ladder-type structure is characterized by a central, four-membered (Sn–O)_2_ ring on both sides extended by six-membered Sn–O–C rings. Its two inner (Sn_*i*_) and two outer (Sn_*o*_) tin atoms are – to a first approximation – fivefold, trigonal–bipyramidally coordinated and linked together *via* two μ_2_-coordinating oxygen atoms (O_*i*_) and two chelating (–COO_*i*_) carboxyl­ate groups. The structure is completed by two monodentate carboxyl­ate groups (–COO_*o*_) attached to the outer tin atoms (Fig. 3 ▸).

The central, planar and centrosymmetric four-membered (Sn–O)_2_ rings exhibit acute [75.90 (5)/75.78 (5)°] angles at tin and obtuse ones [104.10 (5)/104.22 (5)°] at oxygen. Its rhomboidal shape with different Sn—O distances results from the position of the μ_3_-oxygen atom (O1/O3) within the trigonal–bipyramidal coordination sphere of the inner tin atoms (Sn1/Sn3): bonds where the O atom is in an equatorial (*eq*) position are significantly shorter [2.042 (1)/2.046 (1) Å] than those where the O atom is in an axial (*ax*) position [2.164 (1)/2.152 (1) Å]. The second axially positioned Sn—O bond at the inner tin atoms is even longer [2.251 (1)/2.248 (1) Å] as it results from a coordinative bond of the oxygen atom (O11/O31) of the μ_2_-benzoate ligand (–COO_*i*_). In contrast to the prediction of the VSEPR concept, the bond angle between both equatorially positioned *n*-butyl groups is widened to 146.20 (7)/141.73 (7)°.

**Figure 4:**
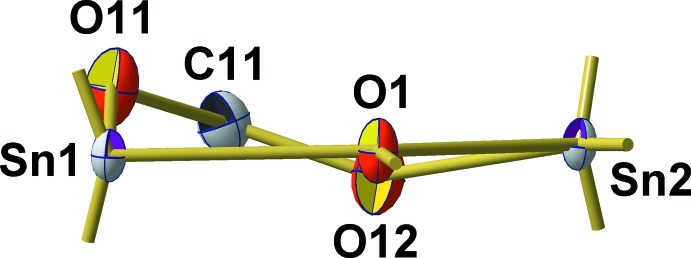
Folded conformation of the outer six-membered Sn–O–C rings of mol­ecule 1 as an example. Displacement ellipsoids are drawn at the 50% probability level and bonds to C atoms are indicated as short sticks.

The conformation of the outer, six-membered Sn–O–C rings is defined by an angle of 19.9 (1)/23.4 (1)° between the O–C–O plane and the Sn–O–Sn plane (Fig. 4 ▸). In case of the trigonal–bipyramidally coordinated outer tin atoms, Sn—O bond lengths follow the rule: *d*(Sn_*o*_—Oμ_3_)_*eq*_ [2.027 (1)/2.022 (1) Å] < *d*(Sn_*o*_—Oμ_1_-carb)_*ax*_ [2.175 (1)/2.188 (1) Å] < *d*(Sn_*o*_—Oμ_2_-carb)_*ax*_ [2.260 (1)/2.235 (1) Å]. Bond angles between the *n*-butyl groups at the tin atoms are 142.49 (7) and 135.64 (7)°.

The different coordination modes of both benzoate ligands are reflected in different C—O bond lengths: in the case of the monodentate carboxyl­ate group (–COO_*o*_), the C—O bonds are of different strengths: the short, strong one [1.232 (2)/1.222 (3) Å] indicates a localized C=O double bond whereas the long, weak one [1.307 (2)/1.306 (3) Å] of the Sn-coordinating oxygen atom indicates a localized C—O single bond. In the case of the bridging benzoate groups (–COO_*i*_) both C—O bonds are of almost equal lengths [1.259 (2),1.258 (2)], in accordance with a delocalized π-system. In the two benzoate ligands, the carboxyl­ate groups and the phenyl groups are not co-planar, but are inclined to each other at angles of 15.1 (2), 14.8 (3)/3.9 (3) and 17.3 (1)°.

**Figure 5:**
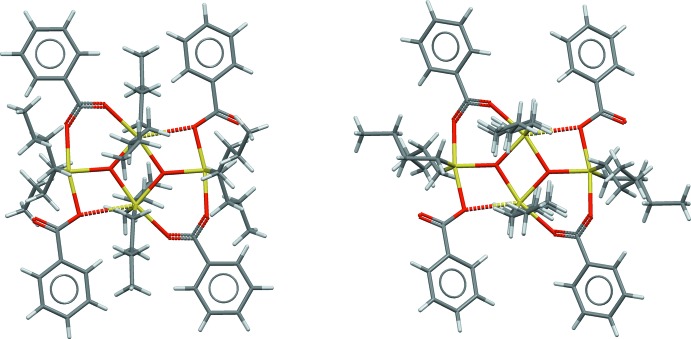
Stick model showing the principal bonding scheme within the two mol­ecules of the title compound, viewed down the central (Sn–O)_2_ ring in order to visualize the different orientations of the *n*-butyl groups with respect to the Sn–O framework of both mol­ecules.

Another characteristic feature of the mol­ecular structure comprises some additional, very weak inter­actions [*d*(Sn⋯O) = 2.7857 (2)/2.7141 (2) Å] of the μ_1_-O atoms (O21/O41) of the outer carboxyl­ate groups with the inner tin atoms (Sn1/Sn3), while those of the μ_0_-O atoms (O22/O42) of the outer carboxyl­ate groups with the outer tin atoms (Sn2/Sn4) are still longer [2.8901 (2)/2.9883 (2) Å]. Taking these weak inter­actions into account, both kinds of Sn atoms adopt a strongly distorted octa­hedral coordination. All bonding features (except the last ones) of the two mol­ecules are summarized in Fig. 5 ▸, which also visualizes the major structural differences between the mol­ecules as a result of the different orientations of the *n*-butyl groups relative to the Sn–O framework.

## Supra­molecular features   

**Figure 6:**
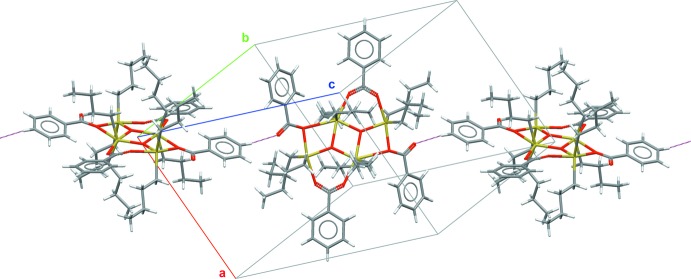
The short, nearly linear, C—H⋯O=C inter­actions (dashed sticks, blue) between two different neighbouring mol­ecules responsible for the chain-like arrangement along the [111] direction.

Inter­molecular inter­actions are restricted to van der Waals and C—H⋯O contacts. Among the latter, a very short [*d*(H⋯O) = 2.487 Å], nearly linear [〈(C—H⋯O) = 172.6°] contact between the hydrogen atom H26 of a phenyl ring of mol­ecule 1 and the non-coordinating oxygen atom O42 of the second mol­ecule attracts attention as it leads to a chain-like arrangement of the two mol­ecules along [111] (Fig. 6 ▸). All other C—H⋯O=C contacts are longer than 2.73 Å.

## Database survey   

Tetraorganodistannoxane di­carboxyl­ates, [*R*_2_Sn(OOC*R*′)]_2_O, have been extensively structurally characterized. The Cambridge Structural Database (Groom *et al.*, 2016 ▸) quotes as many as 214 entries (date: 10.01.2017). The majority of organic moieties attached to tin are found to be *n*-butyl (145) while for the di­carboxyl­ates benzoic acid derivatives (90) are the most studied. Even for the combination of *R* = *n*Bu and *R*′ = benzoic acid derivatives not less than 67 structures are described, but from the parent compound with *R*′ = PhCOO^−^, only the structure of the methyl compound (*R* = Me) has been completely characterized (Amini *et al.*, 2002 ▸).

## Synthesis and crystallization   

[{*n*Bu_2_Sn(OOCPh)}_2_O]_2_ was obtained from an equimolar mixture of 0.300 g (1.2 mmol) of *n*-di­butyl­tin oxide with 0.147 g (1.2 mmol) of benzoic acid in ethanol under reflux for 3.5 h. After removal of the solvent, single crystals were obtained by recrystallization of the solid from ethanol/*n*-hexane.

## Refinement   

Crystal data, data collection and structure refinement details are summarized in Table 2 ▸. Most of the hydrogen atoms were clearly identified in difference Fourier syntheses. Their positions were calculated assuming idealized geometries and allowed to ride on the carbon atoms with C—H = 0.98 Å (–CH_3_), 0.99 Å (–CH_2_–), and 0.95 Å (C—H_arom_) using one common isotropic displacement parameter for each *n*-butyl and phenyl group. Disorder of both *n*-butyl groups at the outer Sn atom (Sn4) of the second mol­ecule was refined using a split model with site occupancies of 0.806 (3)/0.194 (3) and 0.702 (3)/0.298 (3). In order for the structural model to be chemically meaningful, the atomic positions of the minor components were restrained to a target value for the C—C distance [*d*(C—C) = 1.526 (3) Å] and displacement parameters were taken from the chemically equivalent C atoms of the major occupancy component.

## Supplementary Material

Crystal structure: contains datablock(s) I. DOI: 10.1107/S2056989017001505/zl2692sup1.cif

Structure factors: contains datablock(s) I. DOI: 10.1107/S2056989017001505/zl2692Isup2.hkl

CCDC reference: 1530058

Additional supporting information:  crystallographic information; 3D view; checkCIF report

## Figures and Tables

**Table 1:** Selected bond lengths (Å)

Sn1—O1	2.0424 (11)	Sn3—O3	2.0460 (11)
Sn1—C111	2.1335 (19)	Sn3—C311	2.1271 (17)
Sn1—C121	2.1300 (19)	Sn3—C321	2.1268 (18)
Sn1—O1^i^	2.1641 (11)	Sn3—O3^ii^	2.1520 (12)
Sn1—O11	2.2507 (12)	Sn3—O31	2.2475 (13)
Sn2—O1	2.0273 (11)	Sn4—O3	2.0214 (11)
Sn2—C211	2.1342 (19)	Sn4—C411	2.1286 (18)
Sn2—C221	2.1282 (19)	Sn4—C421	2.1266 (19)
Sn2—O21	2.1744 (12)	Sn4—O41	2.1886 (13)
Sn2—O12	2.2601 (12)	Sn4—O32	2.2350 (13)

Symmetry codes: (i) 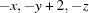; (ii) 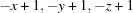.

**Table 2:** Experimental details

Crystal data	
Chemical formula	[Sn_4_(C_4_H_9_)_8_(C_7_H_5_O_2_)_4_O_2_]
*M*_r_	1448.09
Crystal system, space group	Triclinic, *P* 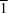
Temperature (K)	100
*a*, *b*, *c* (Å)	14.3221 (6), 14.3742 (6), 17.4049 (7)
α, β, γ (°)	66.915 (2), 81.233 (2), 78.528 (2)
*V* (Å^3^)	3219.3 (2)
*Z*	2
Radiation type	Mo *K*α
μ (mm^−1^)	1.59
Crystal size (mm)	0.42 × 0.26 × 0.22

Data collection	
Diffractometer	Bruker APEXII CCD
Absorption correction	Multi-scan (*SADABS*; Bruker, 2009 ▸)
*T*_min_, *T*_max_	0.556, 0.722
No. of measured, independent and observed [*I* > 2σ(*I*)] reflections	137897, 15505, 13810
*R*_int_	0.035
(sin θ/λ)_max_ (Å^−1^)	0.661

Refinement	
*R*[*F* ^2^ > 2σ(*F* ^2^)], *wR*(*F* ^2^), *S*	0.019, 0.043, 1.04
No. of reflections	15505
No. of parameters	710
No. of restraints	12
H-atom treatment	H-atom parameters constrained
Δρ_max_, Δρ_min_ (e Å^−3^)	0.81, −0.61

Computer programs: *APEX2* and *SAINT* (Bruker, 2009 ▸), *SHELXS97* (Sheldrick, 2008 ▸), *SHELXL2014* (Sheldrick, 2015 ▸), *DIAMOND* (Brandenburg, 2006 ▸), *Mercury* (Macrae *et al.*, 2008 ▸) and *SHELXTL* (Sheldrick, 2008 ▸).

## supplementary crystallographic information

### Crystal data

**Table:**

[Sn_4_(C_4_H_9_)_8_(C_7_H_5_O_2_)_4_O_2_]	*Z* = 2
*M**_r_* = 1448.09	*F*(000) = 1464
Triclinic, *P*1	*D*_x_ = 1.494 Mg m^−^^3^
*a* = 14.3221 (6) Å	Mo *K*α radiation, λ = 0.71073 Å
*b* = 14.3742 (6) Å	Cell parameters from 9485 reflections
*c* = 17.4049 (7) Å	θ = 2.4–28.3°
α = 66.915 (2)°	µ = 1.59 mm^−^^1^
β = 81.233 (2)°	*T* = 100 K
γ = 78.528 (2)°	Parallelepiped, colourless
*V* = 3219.3 (2) Å^3^	0.42 × 0.26 × 0.22 mm

### Data collection

**Table:**

Bruker APEXII CCD diffractometer	13810 reflections with *I* > 2σ(*I*)
φ and ω scans	*R*_int_ = 0.035
Absorption correction: multi-scan (SADABS; Bruker, 2009)	θ_max_ = 28.0°, θ_min_ = 1.9°
*T*_min_ = 0.556, *T*_max_ = 0.722	*h* = −18→18
137897 measured reflections	*k* = −18→18
15505 independent reflections	*l* = −22→22

### Refinement

**Table:**

Refinement on *F*^2^	12 restraints
Least-squares matrix: full	Hydrogen site location: inferred from neighbouring sites
*R*[*F*^2^ > 2σ(*F*^2^)] = 0.019	H-atom parameters constrained
*wR*(*F*^2^) = 0.043	*w* = 1/[σ^2^(*F*_o_^2^) + (0.0156*P*)^2^ + 2.1897*P*] where *P* = (*F*_o_^2^ + 2*F*_c_^2^)/3
*S* = 1.04	(Δ/σ)_max_ = 0.003
15505 reflections	Δρ_max_ = 0.81 e Å^−^^3^
710 parameters	Δρ_min_ = −0.61 e Å^−^^3^

### Special details

**Table:**

Geometry. All esds (except the esd in the dihedral angle between two l.s. planes) are estimated using the full covariance matrix. The cell esds are taken into account individually in the estimation of esds in distances, angles and torsion angles; correlations between esds in cell parameters are only used when they are defined by crystal symmetry. An approximate (isotropic) treatment of cell esds is used for estimating esds involving l.s. planes.

### Fractional atomic coordinates and isotropic or equivalent isotropic displacement parameters (Å^2^)

**Table:**

	*x*	*y*	*z*	*U*_iso_*/*U*_eq_	Occ. (<1)
Sn1	−0.03987 (2)	0.92445 (2)	−0.03511 (2)	0.01446 (3)	
Sn2	0.03972 (2)	0.81339 (2)	0.18341 (2)	0.01445 (3)	
O1	0.01698 (9)	0.91985 (9)	0.06728 (7)	0.0160 (2)	
C111	−0.19099 (13)	0.94730 (15)	−0.00704 (12)	0.0233 (4)	
H111	−0.2031	0.9302	0.0542	0.053 (3)*	
H112	−0.2144	1.0213	−0.0346	0.053 (3)*	
C112	−0.25200 (14)	0.88943 (17)	−0.03089 (13)	0.0284 (4)	
H113	−0.2288	0.8151	−0.0052	0.053 (3)*	
H114	−0.2447	0.9092	−0.0925	0.053 (3)*	
C113	−0.35776 (16)	0.9103 (2)	−0.00266 (16)	0.0420 (6)	
H115	−0.3646	0.8954	0.0583	0.053 (3)*	
H116	−0.3822	0.9838	−0.0316	0.053 (3)*	
C114	−0.4177 (2)	0.8465 (2)	−0.0209 (2)	0.0613 (8)	
H117	−0.4107	0.8604	−0.0812	0.053 (3)*	
H118	−0.4850	0.8641	−0.0035	0.053 (3)*	
H119	−0.3960	0.7737	0.0100	0.053 (3)*	
C121	0.07447 (14)	0.89110 (14)	−0.11872 (11)	0.0208 (4)	
H121	0.0584	0.8403	−0.1379	0.056 (3)*	
H122	0.0818	0.9543	−0.1685	0.056 (3)*	
C122	0.16874 (15)	0.84947 (17)	−0.07840 (13)	0.0295 (4)	
H123	0.1799	0.8959	−0.0523	0.056 (3)*	
H124	0.1635	0.7819	−0.0332	0.056 (3)*	
C123	0.25439 (15)	0.83781 (19)	−0.13868 (14)	0.0357 (5)	
H125	0.2546	0.9027	−0.1880	0.056 (3)*	
H126	0.2481	0.7837	−0.1585	0.056 (3)*	
C124	0.34921 (18)	0.8106 (2)	−0.09965 (19)	0.0550 (7)	
H127	0.4016	0.8008	−0.1404	0.056 (3)*	
H128	0.3490	0.7472	−0.0499	0.056 (3)*	
H129	0.3581	0.8661	−0.0835	0.056 (3)*	
C211	−0.08718 (13)	0.81715 (15)	0.26495 (11)	0.0219 (4)	
H211	−0.1395	0.8575	0.2287	0.048 (2)*	
H212	−0.1013	0.7461	0.2914	0.048 (2)*	
C212	−0.09632 (14)	0.85724 (15)	0.33488 (11)	0.0227 (4)	
H213	−0.0853	0.9294	0.3110	0.048 (2)*	
H214	−0.0471	0.8168	0.3744	0.048 (2)*	
C213	−0.19597 (16)	0.8503 (2)	0.38207 (15)	0.0380 (5)	
H215	−0.2445	0.8888	0.3414	0.048 (2)*	
H216	−0.2057	0.7777	0.4060	0.048 (2)*	
C214	−0.21275 (15)	0.89078 (17)	0.45179 (14)	0.0324 (5)	
H217	−0.1995	0.9613	0.4296	0.048 (2)*	
H218	−0.1702	0.8480	0.4958	0.048 (2)*	
H219	−0.2794	0.8893	0.4752	0.048 (2)*	
C221	0.17341 (14)	0.72163 (14)	0.17206 (12)	0.0222 (4)	
H221	0.2129	0.7640	0.1242	0.045 (2)*	
H222	0.2064	0.7037	0.2232	0.045 (2)*	
C222	0.17112 (16)	0.62290 (15)	0.15960 (13)	0.0284 (4)	
H223	0.1299	0.5808	0.2060	0.045 (2)*	
H224	0.1422	0.6400	0.1066	0.045 (2)*	
C223	0.26983 (18)	0.56040 (18)	0.15659 (15)	0.0411 (6)	
H225	0.3008	0.5474	0.2077	0.045 (2)*	
H226	0.3097	0.6003	0.1076	0.045 (2)*	
C224	0.2651 (2)	0.4579 (2)	0.15053 (19)	0.0641 (9)	
H227	0.2338	0.4704	0.1003	0.064 (3)*	
H228	0.2284	0.4168	0.2004	0.064 (3)*	
H229	0.3300	0.4211	0.1470	0.064 (3)*	
O11	−0.03864 (10)	0.75409 (9)	0.01683 (8)	0.0241 (3)	
O12	−0.03990 (10)	0.71155 (10)	0.15460 (8)	0.0236 (3)	
C11	−0.04706 (13)	0.69199 (13)	0.09152 (11)	0.0196 (4)	
C12	−0.06755 (14)	0.58777 (14)	0.10684 (12)	0.0237 (4)	
C13	−0.09874 (17)	0.56773 (16)	0.04403 (14)	0.0346 (5)	
H13	−0.1063	0.6199	−0.0099	0.047 (3)*	
C14	−0.1189 (2)	0.47041 (19)	0.06069 (16)	0.0492 (7)	
H14	−0.1411	0.4564	0.0182	0.047 (3)*	
C15	−0.1068 (2)	0.39439 (18)	0.13877 (17)	0.0482 (7)	
H15	−0.1205	0.3282	0.1497	0.047 (3)*	
C16	−0.0750 (2)	0.41364 (17)	0.20105 (16)	0.0439 (6)	
H16	−0.0659	0.3607	0.2544	0.047 (3)*	
C17	−0.05638 (17)	0.51081 (15)	0.18543 (14)	0.0329 (5)	
H17	−0.0359	0.5248	0.2287	0.047 (3)*	
O21	0.09580 (9)	0.93884 (9)	0.19155 (7)	0.0191 (3)	
O22	0.13977 (9)	0.81985 (9)	0.31306 (8)	0.0213 (3)	
C21	0.13446 (12)	0.90883 (14)	0.26235 (11)	0.0172 (3)	
C22	0.17197 (13)	0.98877 (14)	0.27895 (11)	0.0179 (4)	
C23	0.14682 (14)	1.09279 (14)	0.23253 (12)	0.0219 (4)	
H23	0.1055	1.1143	0.1889	0.030 (3)*	
C24	0.18210 (15)	1.16491 (16)	0.24995 (13)	0.0290 (4)	
H24	0.1648	1.2357	0.2183	0.030 (3)*	
C25	0.24249 (15)	1.13388 (17)	0.31330 (14)	0.0318 (5)	
H25	0.2669	1.1834	0.3249	0.030 (3)*	
C26	0.26727 (15)	1.03149 (17)	0.35949 (14)	0.0307 (5)	
H26	0.3087	1.0107	0.4030	0.030 (3)*	
C27	0.23215 (13)	0.95773 (16)	0.34305 (12)	0.0231 (4)	
H27	0.2492	0.8871	0.3753	0.030 (3)*	
Sn3	0.61120 (2)	0.44791 (2)	0.52321 (2)	0.01354 (3)	
Sn4	0.49447 (2)	0.36021 (2)	0.38939 (2)	0.01504 (3)	
O3	0.50553 (8)	0.43974 (9)	0.46018 (7)	0.0150 (2)	
C311	0.60956 (13)	0.34279 (13)	0.64978 (11)	0.0186 (4)	
H311	0.6765	0.3155	0.6639	0.038 (2)*	
H312	0.5800	0.3807	0.6864	0.038 (2)*	
C312	0.55676 (14)	0.25282 (14)	0.67031 (11)	0.0215 (4)	
H313	0.5861	0.2136	0.6347	0.038 (2)*	
H314	0.4893	0.2789	0.6574	0.038 (2)*	
C313	0.55999 (17)	0.18205 (15)	0.76230 (12)	0.0303 (5)	
H315	0.6275	0.1563	0.7750	0.038 (2)*	
H316	0.5308	0.2216	0.7978	0.038 (2)*	
C314	0.5076 (2)	0.09152 (19)	0.78418 (15)	0.0478 (7)	
H317	0.5082	0.0509	0.8444	0.038 (2)*	
H318	0.5396	0.0489	0.7527	0.038 (2)*	
H319	0.4414	0.1165	0.7695	0.038 (2)*	
C321	0.69759 (14)	0.54809 (15)	0.42927 (12)	0.0239 (4)	
H321	0.6832	0.6157	0.4345	0.036 (4)*	
H322	0.7656	0.5210	0.4397	0.036 (4)*	
C322	0.68403 (14)	0.56270 (14)	0.34011 (11)	0.0216 (4)	
H323	0.6148	0.5759	0.3329	0.042 (3)*	
H324	0.7117	0.4988	0.3307	0.042 (3)*	
C323	0.73061 (15)	0.65076 (16)	0.27503 (13)	0.0293 (4)	
H325	0.7083	0.7131	0.2881	0.042 (3)*	
H326	0.8008	0.6341	0.2779	0.042 (3)*	
C324	0.70742 (17)	0.6723 (2)	0.18683 (14)	0.0442 (6)	
H327	0.6382	0.6914	0.1831	0.042 (3)*	
H328	0.7295	0.6108	0.1735	0.042 (3)*	
H329	0.7396	0.7286	0.1469	0.042 (3)*	
O31	0.69881 (10)	0.31322 (10)	0.49789 (8)	0.0261 (3)	
O32	0.65313 (9)	0.31492 (10)	0.38033 (8)	0.0201 (3)	
C31	0.71303 (13)	0.28987 (13)	0.43385 (11)	0.0173 (3)	
C32	0.80661 (12)	0.22829 (13)	0.42121 (11)	0.0162 (3)	
C33	0.87661 (13)	0.20435 (14)	0.47628 (11)	0.0205 (4)	
H33	0.8650	0.2278	0.5214	0.028 (3)*	
C34	0.96322 (14)	0.14621 (15)	0.46514 (13)	0.0260 (4)	
H34	1.0107	0.1292	0.5031	0.028 (3)*	
C35	0.98102 (14)	0.11283 (15)	0.39920 (12)	0.0251 (4)	
H35	1.0408	0.0733	0.3917	0.028 (3)*	
C36	0.91186 (14)	0.13692 (14)	0.34414 (12)	0.0238 (4)	
H36	0.9243	0.1141	0.2987	0.028 (3)*	
C37	0.82457 (13)	0.19424 (13)	0.35504 (11)	0.0192 (4)	
H37	0.7770	0.2103	0.3173	0.028 (3)*	
O41	0.34751 (9)	0.43755 (10)	0.39771 (8)	0.0209 (3)	
O42	0.30888 (10)	0.32399 (11)	0.35606 (10)	0.0320 (3)	
C41	0.28552 (14)	0.39698 (14)	0.37840 (12)	0.0214 (4)	
C42	0.18298 (13)	0.44420 (14)	0.38510 (11)	0.0206 (4)	
C43	0.15777 (13)	0.54131 (14)	0.38792 (11)	0.0209 (4)	
H43	0.2063	0.5783	0.3870	0.032 (3)*	
C44	0.06256 (14)	0.58483 (15)	0.39207 (12)	0.0253 (4)	
H44	0.0459	0.6517	0.3931	0.032 (3)*	
C45	−0.00816 (14)	0.53014 (16)	0.39474 (12)	0.0303 (5)	
H45	−0.0735	0.5596	0.3980	0.032 (3)*	
C46	0.01560 (15)	0.43340 (16)	0.39270 (13)	0.0313 (5)	
H46	−0.0334	0.3960	0.3955	0.032 (3)*	
C47	0.11084 (15)	0.39030 (16)	0.38658 (13)	0.0279 (4)	
H47	0.1271	0.3245	0.3834	0.032 (3)*	
C411	0.47723 (15)	0.20853 (14)	0.47207 (12)	0.0259 (4)	0.806 (3)
H411	0.5322	0.1788	0.5070	0.032 (2)*	0.806 (3)
H412	0.4186	0.2113	0.5100	0.032 (2)*	0.806 (3)
C412	0.47008 (15)	0.13829 (16)	0.42666 (14)	0.0222 (5)	0.806 (3)
H413	0.4514	0.0732	0.4682	0.032 (2)*	0.806 (3)
H414	0.4195	0.1712	0.3873	0.032 (2)*	0.806 (3)
C413	0.56410 (18)	0.11528 (19)	0.37830 (17)	0.0267 (6)	0.806 (3)
H415	0.5830	0.1801	0.3365	0.032 (2)*	0.806 (3)
H416	0.6149	0.0818	0.4175	0.032 (2)*	0.806 (3)
C414	0.5540 (4)	0.0455 (5)	0.3339 (5)	0.0358 (11)	0.806 (3)
H417	0.5369	−0.0194	0.3755	0.032 (2)*	0.806 (3)
H418	0.6148	0.0322	0.3026	0.032 (2)*	0.806 (3)
H419	0.5038	0.0787	0.2950	0.032 (2)*	0.806 (3)
C415	0.47723 (15)	0.20853 (14)	0.47207 (12)	0.0259 (4)	0.194 (3)
H431	0.4871	0.1996	0.5298	0.032 (2)*	0.194 (3)
H432	0.4112	0.1976	0.4720	0.032 (2)*	0.194 (3)
C416	0.5483 (5)	0.1284 (6)	0.4462 (5)	0.0222 (5)	0.194 (3)
H433	0.6136	0.1465	0.4355	0.032 (2)*	0.194 (3)
H434	0.5491	0.0601	0.4917	0.032 (2)*	0.194 (3)
C417	0.5168 (9)	0.1268 (9)	0.3670 (6)	0.0267 (6)	0.194 (3)
H435	0.4497	0.1143	0.3788	0.032 (2)*	0.194 (3)
H436	0.5174	0.1960	0.3230	0.032 (2)*	0.194 (3)
C418	0.5738 (19)	0.050 (2)	0.331 (2)	0.0358 (11)	0.194 (3)
H437	0.5589	−0.0187	0.3653	0.032 (2)*	0.194 (3)
H438	0.6423	0.0503	0.3296	0.032 (2)*	0.194 (3)
H439	0.5569	0.0686	0.2734	0.032 (2)*	0.194 (3)
C421	0.50793 (14)	0.43918 (16)	0.25721 (12)	0.0288 (4)	0.702 (3)
H421	0.4501	0.4346	0.2349	0.095 (5)*	0.702 (3)
H422	0.5069	0.5123	0.2462	0.095 (5)*	0.702 (3)
C422	0.5947 (2)	0.4060 (3)	0.20591 (16)	0.0399 (8)	0.702 (3)
H423	0.6515	0.4258	0.2182	0.095 (5)*	0.702 (3)
H424	0.6047	0.3303	0.2259	0.095 (5)*	0.702 (3)
C423	0.5910 (4)	0.4481 (3)	0.11158 (18)	0.0542 (11)	0.702 (3)
H425	0.6524	0.4229	0.0863	0.095 (5)*	0.702 (3)
H426	0.5400	0.4206	0.0983	0.095 (5)*	0.702 (3)
C424	0.5727 (4)	0.5628 (3)	0.0715 (3)	0.0704 (13)	0.702 (3)
H427	0.5133	0.5890	0.0975	0.095 (5)*	0.702 (3)
H428	0.5671	0.5834	0.0114	0.095 (5)*	0.702 (3)
H429	0.6260	0.5908	0.0794	0.095 (5)*	0.702 (3)
C425	0.50793 (14)	0.43918 (16)	0.25721 (12)	0.0288 (4)	0.298 (3)
H441	0.4477	0.4868	0.2411	0.095 (5)*	0.298 (3)
H442	0.5593	0.4815	0.2439	0.095 (5)*	0.298 (3)
C426	0.5292 (7)	0.3773 (4)	0.2019 (3)	0.0399 (8)	0.298 (3)
H443	0.4798	0.3328	0.2146	0.095 (5)*	0.298 (3)
H444	0.5921	0.3332	0.2129	0.095 (5)*	0.298 (3)
C427	0.5303 (7)	0.4492 (8)	0.1100 (4)	0.0542 (11)	0.298 (3)
H445	0.5197	0.4114	0.0760	0.095 (5)*	0.298 (3)
H446	0.4764	0.5061	0.1036	0.095 (5)*	0.298 (3)
C428	0.6228 (7)	0.4941 (10)	0.0753 (7)	0.0704 (13)	0.298 (3)
H447	0.6742	0.4404	0.0696	0.095 (5)*	0.298 (3)
H448	0.6398	0.5217	0.1138	0.095 (5)*	0.298 (3)
H449	0.6139	0.5491	0.0204	0.095 (5)*	0.298 (3)

### Atomic displacement parameters (Å^2^)

**Table:**

	*U*^11^	*U*^22^	*U*^33^	*U*^12^	*U*^13^	*U*^23^
Sn1	0.01998 (6)	0.01333 (6)	0.01166 (5)	−0.00403 (4)	−0.00460 (4)	−0.00444 (4)
Sn2	0.01999 (6)	0.01252 (5)	0.01096 (5)	−0.00310 (4)	−0.00446 (4)	−0.00309 (4)
O1	0.0237 (7)	0.0134 (6)	0.0119 (6)	−0.0047 (5)	−0.0060 (5)	−0.0034 (5)
C111	0.0221 (10)	0.0275 (10)	0.0200 (9)	−0.0060 (8)	−0.0013 (7)	−0.0077 (8)
C112	0.0265 (11)	0.0358 (11)	0.0240 (10)	−0.0126 (9)	−0.0039 (8)	−0.0080 (9)
C113	0.0271 (12)	0.0532 (15)	0.0417 (14)	−0.0121 (11)	−0.0038 (10)	−0.0104 (12)
C114	0.0381 (15)	0.0655 (19)	0.073 (2)	−0.0225 (14)	−0.0170 (14)	−0.0069 (16)
C121	0.0288 (10)	0.0183 (9)	0.0146 (8)	−0.0013 (7)	−0.0036 (7)	−0.0058 (7)
C122	0.0271 (11)	0.0399 (12)	0.0250 (10)	0.0014 (9)	−0.0049 (8)	−0.0181 (9)
C123	0.0273 (11)	0.0444 (13)	0.0343 (12)	−0.0001 (10)	−0.0003 (9)	−0.0170 (10)
C124	0.0310 (14)	0.075 (2)	0.0684 (19)	0.0012 (13)	−0.0057 (13)	−0.0405 (17)
C211	0.0224 (10)	0.0254 (10)	0.0177 (9)	−0.0050 (8)	−0.0014 (7)	−0.0073 (8)
C212	0.0257 (10)	0.0232 (9)	0.0180 (9)	−0.0020 (8)	−0.0018 (7)	−0.0072 (8)
C213	0.0255 (11)	0.0577 (15)	0.0383 (13)	−0.0062 (10)	0.0007 (9)	−0.0273 (12)
C214	0.0274 (11)	0.0375 (12)	0.0326 (11)	−0.0053 (9)	0.0045 (9)	−0.0160 (10)
C221	0.0241 (10)	0.0206 (9)	0.0194 (9)	0.0009 (7)	−0.0059 (7)	−0.0055 (7)
C222	0.0387 (12)	0.0238 (10)	0.0234 (10)	0.0078 (9)	−0.0146 (9)	−0.0113 (8)
C223	0.0493 (15)	0.0375 (13)	0.0340 (12)	0.0182 (11)	−0.0159 (11)	−0.0179 (10)
C224	0.093 (2)	0.0501 (16)	0.0583 (18)	0.0388 (16)	−0.0441 (17)	−0.0395 (15)
O11	0.0392 (8)	0.0160 (6)	0.0186 (6)	−0.0080 (6)	−0.0065 (6)	−0.0047 (5)
O12	0.0336 (8)	0.0209 (7)	0.0199 (7)	−0.0118 (6)	−0.0041 (6)	−0.0070 (5)
C11	0.0216 (9)	0.0169 (8)	0.0217 (9)	−0.0055 (7)	−0.0041 (7)	−0.0067 (7)
C12	0.0298 (11)	0.0181 (9)	0.0253 (10)	−0.0091 (8)	−0.0034 (8)	−0.0074 (8)
C13	0.0539 (15)	0.0278 (11)	0.0285 (11)	−0.0166 (10)	−0.0072 (10)	−0.0109 (9)
C14	0.080 (2)	0.0385 (13)	0.0453 (15)	−0.0297 (13)	−0.0080 (14)	−0.0217 (12)
C15	0.0745 (19)	0.0235 (11)	0.0524 (16)	−0.0250 (12)	−0.0031 (14)	−0.0127 (11)
C16	0.0651 (17)	0.0221 (11)	0.0421 (14)	−0.0184 (11)	−0.0102 (12)	−0.0016 (10)
C17	0.0469 (14)	0.0218 (10)	0.0301 (11)	−0.0144 (9)	−0.0080 (10)	−0.0038 (9)
O21	0.0271 (7)	0.0183 (6)	0.0144 (6)	−0.0064 (5)	−0.0074 (5)	−0.0054 (5)
O22	0.0281 (7)	0.0194 (6)	0.0166 (6)	−0.0047 (5)	−0.0065 (5)	−0.0048 (5)
C21	0.0166 (9)	0.0220 (9)	0.0156 (8)	−0.0042 (7)	−0.0017 (7)	−0.0089 (7)
C22	0.0177 (9)	0.0240 (9)	0.0165 (8)	−0.0067 (7)	0.0020 (7)	−0.0120 (7)
C23	0.0242 (10)	0.0243 (9)	0.0207 (9)	−0.0084 (8)	−0.0006 (7)	−0.0105 (8)
C24	0.0317 (11)	0.0271 (10)	0.0338 (11)	−0.0112 (9)	0.0029 (9)	−0.0163 (9)
C25	0.0280 (11)	0.0412 (12)	0.0427 (13)	−0.0153 (9)	0.0014 (9)	−0.0301 (11)
C26	0.0251 (11)	0.0449 (13)	0.0345 (11)	−0.0078 (9)	−0.0076 (9)	−0.0248 (10)
C27	0.0208 (9)	0.0311 (10)	0.0225 (9)	−0.0050 (8)	−0.0029 (7)	−0.0144 (8)
Sn3	0.01207 (6)	0.01606 (6)	0.01332 (5)	−0.00164 (4)	−0.00290 (4)	−0.00592 (4)
Sn4	0.01779 (6)	0.01416 (6)	0.01564 (6)	−0.00312 (4)	−0.00384 (4)	−0.00697 (5)
O3	0.0146 (6)	0.0162 (6)	0.0170 (6)	−0.0018 (5)	−0.0040 (5)	−0.0085 (5)
C311	0.0227 (9)	0.0187 (9)	0.0153 (8)	−0.0010 (7)	−0.0057 (7)	−0.0069 (7)
C312	0.0264 (10)	0.0192 (9)	0.0189 (9)	−0.0037 (7)	−0.0028 (7)	−0.0067 (7)
C313	0.0423 (13)	0.0243 (10)	0.0216 (10)	−0.0065 (9)	−0.0002 (9)	−0.0060 (8)
C314	0.0714 (19)	0.0361 (13)	0.0316 (13)	−0.0229 (13)	0.0036 (12)	−0.0042 (11)
C321	0.0213 (10)	0.0327 (11)	0.0208 (9)	−0.0136 (8)	0.0007 (7)	−0.0097 (8)
C322	0.0214 (9)	0.0251 (10)	0.0193 (9)	−0.0082 (8)	−0.0011 (7)	−0.0073 (8)
C323	0.0277 (11)	0.0298 (11)	0.0259 (10)	−0.0116 (9)	0.0004 (8)	−0.0032 (9)
C324	0.0353 (13)	0.0579 (16)	0.0235 (11)	−0.0136 (12)	−0.0021 (10)	0.0048 (11)
O31	0.0272 (7)	0.0295 (7)	0.0210 (7)	0.0107 (6)	−0.0070 (6)	−0.0143 (6)
O32	0.0182 (6)	0.0224 (6)	0.0202 (6)	−0.0002 (5)	−0.0036 (5)	−0.0093 (5)
C31	0.0192 (9)	0.0150 (8)	0.0158 (8)	−0.0025 (7)	−0.0011 (7)	−0.0038 (7)
C32	0.0181 (9)	0.0128 (8)	0.0161 (8)	−0.0022 (7)	−0.0003 (7)	−0.0041 (7)
C33	0.0217 (9)	0.0203 (9)	0.0198 (9)	−0.0014 (7)	−0.0026 (7)	−0.0086 (7)
C34	0.0191 (10)	0.0298 (10)	0.0266 (10)	0.0012 (8)	−0.0041 (8)	−0.0094 (8)
C35	0.0207 (10)	0.0221 (9)	0.0278 (10)	0.0022 (8)	0.0040 (8)	−0.0090 (8)
C36	0.0299 (11)	0.0203 (9)	0.0208 (9)	−0.0019 (8)	0.0045 (8)	−0.0107 (8)
C37	0.0228 (9)	0.0169 (8)	0.0182 (9)	−0.0041 (7)	−0.0008 (7)	−0.0066 (7)
O41	0.0168 (6)	0.0246 (7)	0.0268 (7)	−0.0036 (5)	−0.0066 (5)	−0.0135 (6)
O42	0.0298 (8)	0.0325 (8)	0.0454 (9)	−0.0019 (6)	−0.0139 (7)	−0.0245 (7)
C41	0.0232 (10)	0.0228 (9)	0.0212 (9)	−0.0064 (8)	−0.0069 (7)	−0.0080 (8)
C42	0.0197 (9)	0.0254 (9)	0.0168 (9)	−0.0071 (7)	−0.0066 (7)	−0.0042 (7)
C43	0.0197 (9)	0.0256 (9)	0.0168 (9)	−0.0069 (7)	−0.0041 (7)	−0.0046 (7)
C44	0.0228 (10)	0.0261 (10)	0.0204 (9)	−0.0020 (8)	−0.0049 (8)	−0.0014 (8)
C45	0.0180 (10)	0.0374 (12)	0.0241 (10)	−0.0065 (8)	−0.0044 (8)	0.0027 (9)
C46	0.0235 (10)	0.0351 (12)	0.0278 (11)	−0.0155 (9)	−0.0101 (8)	0.0043 (9)
C47	0.0298 (11)	0.0254 (10)	0.0274 (10)	−0.0118 (8)	−0.0106 (8)	−0.0023 (8)
C411	0.0369 (12)	0.0195 (9)	0.0213 (9)	−0.0084 (8)	0.0026 (8)	−0.0075 (8)
C412	0.0230 (12)	0.0159 (11)	0.0273 (12)	−0.0038 (9)	−0.0029 (9)	−0.0071 (9)
C413	0.0244 (15)	0.0209 (11)	0.0358 (14)	−0.0054 (12)	0.0020 (12)	−0.0124 (10)
C414	0.044 (3)	0.0282 (14)	0.0374 (14)	−0.0106 (18)	0.008 (2)	−0.0167 (12)
C415	0.0369 (12)	0.0195 (9)	0.0213 (9)	−0.0084 (8)	0.0026 (8)	−0.0075 (8)
C416	0.0230 (12)	0.0159 (11)	0.0273 (12)	−0.0038 (9)	−0.0029 (9)	−0.0071 (9)
C417	0.0244 (15)	0.0209 (11)	0.0358 (14)	−0.0054 (12)	0.0020 (12)	−0.0124 (10)
C418	0.044 (3)	0.0282 (14)	0.0374 (14)	−0.0106 (18)	0.008 (2)	−0.0167 (12)
C421	0.0311 (11)	0.0333 (11)	0.0175 (9)	0.0016 (9)	−0.0070 (8)	−0.0061 (8)
C422	0.051 (2)	0.0460 (18)	0.0179 (13)	−0.0056 (15)	0.0014 (14)	−0.0094 (13)
C423	0.066 (3)	0.067 (2)	0.0215 (15)	−0.005 (3)	−0.002 (2)	−0.0109 (15)
C424	0.106 (4)	0.060 (3)	0.040 (2)	−0.008 (2)	−0.009 (2)	−0.015 (2)
C425	0.0311 (11)	0.0333 (11)	0.0175 (9)	0.0016 (9)	−0.0070 (8)	−0.0061 (8)
C426	0.051 (2)	0.0460 (18)	0.0179 (13)	−0.0056 (15)	0.0014 (14)	−0.0094 (13)
C427	0.066 (3)	0.067 (2)	0.0215 (15)	−0.005 (3)	−0.002 (2)	−0.0109 (15)
C428	0.106 (4)	0.060 (3)	0.040 (2)	−0.008 (2)	−0.009 (2)	−0.015 (2)

### Geometric parameters (Å, º)

**Table:**

Sn1—O1	2.0424 (11)	C311—H312	0.9900
Sn1—C111	2.1335 (19)	C312—C313	1.525 (3)
Sn1—C121	2.1300 (19)	C312—H313	0.9900
Sn1—O1^i^	2.1641 (11)	C312—H314	0.9900
Sn1—O11	2.2507 (12)	C313—C314	1.521 (3)
Sn2—O1	2.0273 (11)	C313—H315	0.9900
Sn2—C211	2.1342 (19)	C313—H316	0.9900
Sn2—C221	2.1282 (19)	C314—H317	0.9800
Sn2—O21	2.1744 (12)	C314—H318	0.9800
Sn2—O12	2.2601 (12)	C314—H319	0.9800
O1—Sn1^i^	2.1641 (11)	C321—C322	1.520 (2)
C111—C112	1.516 (3)	C321—H321	0.9900
C111—H111	0.9900	C321—H322	0.9900
C111—H112	0.9900	C322—C323	1.520 (3)
C112—C113	1.528 (3)	C322—H323	0.9900
C112—H113	0.9900	C322—H324	0.9900
C112—H114	0.9900	C323—C324	1.518 (3)
C113—C114	1.519 (3)	C323—H325	0.9900
C113—H115	0.9900	C323—H326	0.9900
C113—H116	0.9900	C324—H327	0.9800
C114—H117	0.9800	C324—H328	0.9800
C114—H118	0.9800	C324—H329	0.9800
C114—H119	0.9800	O31—C31	1.262 (2)
C121—C122	1.520 (3)	O32—C31	1.261 (2)
C121—H121	0.9900	C31—C32	1.490 (2)
C121—H122	0.9900	C32—C37	1.391 (2)
C122—C123	1.516 (3)	C32—C33	1.393 (2)
C122—H123	0.9900	C33—C34	1.384 (3)
C122—H124	0.9900	C33—H33	0.9500
C123—C124	1.523 (3)	C34—C35	1.381 (3)
C123—H125	0.9900	C34—H34	0.9500
C123—H126	0.9900	C35—C36	1.383 (3)
C124—H127	0.9800	C35—H35	0.9500
C124—H128	0.9800	C36—C37	1.385 (3)
C124—H129	0.9800	C36—H36	0.9500
C211—C212	1.517 (2)	C37—H37	0.9500
C211—H211	0.9900	O41—C41	1.301 (2)
C211—H212	0.9900	O42—C41	1.226 (2)
C212—C213	1.535 (3)	C41—C42	1.498 (3)
C212—H213	0.9900	C42—C43	1.389 (3)
C212—H214	0.9900	C42—C47	1.401 (3)
C213—C214	1.510 (3)	C43—C44	1.386 (3)
C213—H215	0.9900	C43—H43	0.9500
C213—H216	0.9900	C44—C45	1.386 (3)
C214—H217	0.9800	C44—H44	0.9500
C214—H218	0.9800	C45—C46	1.377 (3)
C214—H219	0.9800	C45—H45	0.9500
C221—C222	1.525 (3)	C46—C47	1.388 (3)
C221—H221	0.9900	C46—H46	0.9500
C221—H222	0.9900	C47—H47	0.9500
C222—C223	1.523 (3)	C411—C412	1.532 (2)
C222—H223	0.9900	C411—H411	0.9900
C222—H224	0.9900	C411—H412	0.9900
C223—C224	1.532 (3)	C412—C413	1.525 (2)
C223—H225	0.9900	C412—H413	0.9900
C223—H226	0.9900	C412—H414	0.9900
C224—H227	0.9800	C413—C414	1.525 (3)
C224—H228	0.9800	C413—H415	0.9900
C224—H229	0.9800	C413—H416	0.9900
O11—C11	1.259 (2)	C414—H417	0.9800
O12—C11	1.258 (2)	C414—H418	0.9800
C11—C12	1.496 (2)	C414—H419	0.9800
C12—C13	1.387 (3)	Sn4—C415	2.1285 (18)
C12—C17	1.389 (3)	C415—C416	1.538 (3)
C13—C14	1.394 (3)	C415—H431	0.9900
C13—H13	0.9500	C415—H432	0.9900
C14—C15	1.379 (4)	C416—C417	1.525 (3)
C14—H14	0.9500	C416—H433	0.9900
C15—C16	1.377 (3)	C416—H434	0.9900
C15—H15	0.9500	C417—C418	1.524 (3)
C16—C17	1.387 (3)	C417—H435	0.9900
C16—H16	0.9500	C417—H436	0.9900
C17—H17	0.9500	C418—H437	0.9800
O21—C21	1.307 (2)	C418—H438	0.9800
O22—C21	1.232 (2)	C418—H439	0.9800
C21—C22	1.498 (2)	C421—C422	1.526 (2)
C22—C27	1.392 (2)	C421—H421	0.9900
C22—C23	1.395 (3)	C421—H422	0.9900
C23—C24	1.387 (3)	C422—C423	1.516 (3)
C23—H23	0.9500	C422—H423	0.9900
C24—C25	1.384 (3)	C422—H424	0.9900
C24—H24	0.9500	C423—C424	1.501 (3)
C25—C26	1.375 (3)	C423—H425	0.9900
C25—H25	0.9500	C423—H426	0.9900
C26—C27	1.397 (3)	C424—H427	0.9800
C26—H26	0.9500	C424—H428	0.9800
C27—H27	0.9500	C424—H429	0.9800
Sn3—O3	2.0460 (11)	Sn4—C425	2.1266 (19)
Sn3—C311	2.1271 (17)	C425—C426	1.512 (3)
Sn3—C321	2.1268 (18)	C425—H441	0.9900
Sn3—O3^ii^	2.1520 (12)	C425—H442	0.9900
Sn3—O31	2.2475 (13)	C426—C427	1.526 (3)
Sn4—O3	2.0214 (11)	C426—H443	0.9900
Sn4—C411	2.1286 (18)	C426—H444	0.9900
Sn4—C421	2.1266 (19)	C427—C428	1.526 (3)
Sn4—O41	2.1886 (13)	C427—H445	0.9900
Sn4—O32	2.2350 (13)	C427—H446	0.9900
O3—Sn3^ii^	2.1521 (12)	C428—H447	0.9800
C311—C312	1.522 (2)	C428—H448	0.9800
C311—H311	0.9900	C428—H449	0.9800
			
O1—Sn1—C121	108.22 (6)	C312—C311—H312	108.3
O1—Sn1—C111	105.19 (6)	Sn3—C311—H312	108.3
C121—Sn1—C111	146.21 (7)	H311—C311—H312	107.4
O1—Sn1—O1^i^	75.90 (5)	C311—C312—C313	111.66 (15)
C121—Sn1—O1^i^	95.08 (6)	C311—C312—H313	109.3
C111—Sn1—O1^i^	97.77 (6)	C313—C312—H313	109.3
O1—Sn1—O11	92.83 (5)	C311—C312—H314	109.3
C121—Sn1—O11	83.57 (6)	C313—C312—H314	109.3
C111—Sn1—O11	89.94 (6)	H313—C312—H314	107.9
O1^i^—Sn1—O11	167.66 (5)	C314—C313—C312	112.54 (18)
O1—Sn2—C221	106.53 (6)	C314—C313—H315	109.1
O1—Sn2—C211	109.62 (6)	C312—C313—H315	109.1
C221—Sn2—C211	142.50 (7)	C314—C313—H316	109.1
O1—Sn2—O21	80.33 (4)	C312—C313—H316	109.1
C221—Sn2—O21	97.30 (6)	H315—C313—H316	107.8
C211—Sn2—O21	98.22 (6)	C313—C314—H317	109.5
O1—Sn2—O12	88.40 (5)	C313—C314—H318	109.5
C221—Sn2—O12	91.80 (6)	H317—C314—H318	109.5
C211—Sn2—O12	79.66 (6)	C313—C314—H319	109.5
O21—Sn2—O12	167.14 (5)	H317—C314—H319	109.5
Sn2—O1—Sn1	135.12 (6)	H318—C314—H319	109.5
Sn2—O1—Sn1^i^	120.42 (5)	C322—C321—Sn3	114.50 (12)
Sn1—O1—Sn1^i^	104.10 (5)	C322—C321—H321	108.6
C112—C111—Sn1	119.14 (14)	Sn3—C321—H321	108.6
C112—C111—H111	107.5	C322—C321—H322	108.6
Sn1—C111—H111	107.5	Sn3—C321—H322	108.6
C112—C111—H112	107.5	H321—C321—H322	107.6
Sn1—C111—H112	107.5	C321—C322—C323	112.53 (16)
H111—C111—H112	107.0	C321—C322—H323	109.1
C111—C112—C113	112.58 (18)	C323—C322—H323	109.1
C111—C112—H113	109.1	C321—C322—H324	109.1
C113—C112—H113	109.1	C323—C322—H324	109.1
C111—C112—H114	109.1	H323—C322—H324	107.8
C113—C112—H114	109.1	C324—C323—C322	112.11 (18)
H113—C112—H114	107.8	C324—C323—H325	109.2
C114—C113—C112	112.5 (2)	C322—C323—H325	109.2
C114—C113—H115	109.1	C324—C323—H326	109.2
C112—C113—H115	109.1	C322—C323—H326	109.2
C114—C113—H116	109.1	H325—C323—H326	107.9
C112—C113—H116	109.1	C323—C324—H327	109.5
H115—C113—H116	107.8	C323—C324—H328	109.5
C113—C114—H117	109.5	H327—C324—H328	109.5
C113—C114—H118	109.5	C323—C324—H329	109.5
H117—C114—H118	109.5	H327—C324—H329	109.5
C113—C114—H119	109.5	H328—C324—H329	109.5
H117—C114—H119	109.5	C31—O31—Sn3	132.36 (12)
H118—C114—H119	109.5	C31—O32—Sn4	131.00 (12)
C122—C121—Sn1	112.48 (12)	O32—C31—O31	124.28 (17)
C122—C121—H121	109.1	O32—C31—C32	118.13 (15)
Sn1—C121—H121	109.1	O31—C31—C32	117.59 (15)
C122—C121—H122	109.1	C37—C32—C33	119.71 (17)
Sn1—C121—H122	109.1	C37—C32—C31	120.57 (16)
H121—C121—H122	107.8	C33—C32—C31	119.72 (16)
C123—C122—C121	114.20 (17)	C34—C33—C32	119.78 (17)
C123—C122—H123	108.7	C34—C33—H33	120.1
C121—C122—H123	108.7	C32—C33—H33	120.1
C123—C122—H124	108.7	C35—C34—C33	120.40 (18)
C121—C122—H124	108.7	C35—C34—H34	119.8
H123—C122—H124	107.6	C33—C34—H34	119.8
C122—C123—C124	113.1 (2)	C34—C35—C36	119.97 (18)
C122—C123—H125	109.0	C34—C35—H35	120.0
C124—C123—H125	109.0	C36—C35—H35	120.0
C122—C123—H126	109.0	C35—C36—C37	120.20 (17)
C124—C123—H126	109.0	C35—C36—H36	119.9
H125—C123—H126	107.8	C37—C36—H36	119.9
C123—C124—H127	109.5	C36—C37—C32	119.94 (17)
C123—C124—H128	109.5	C36—C37—H37	120.0
H127—C124—H128	109.5	C32—C37—H37	120.0
C123—C124—H129	109.5	C41—O41—Sn4	113.34 (11)
H127—C124—H129	109.5	O42—C41—O41	122.39 (18)
H128—C124—H129	109.5	O42—C41—C42	121.34 (16)
C212—C211—Sn2	123.08 (13)	O41—C41—C42	116.27 (16)
C212—C211—H211	106.6	C43—C42—C47	119.17 (18)
Sn2—C211—H211	106.6	C43—C42—C41	121.47 (16)
C212—C211—H212	106.6	C47—C42—C41	119.35 (17)
Sn2—C211—H212	106.6	C44—C43—C42	120.69 (17)
H211—C211—H212	106.5	C44—C43—H43	119.7
C211—C212—C213	110.73 (16)	C42—C43—H43	119.7
C211—C212—H213	109.5	C45—C44—C43	119.58 (19)
C213—C212—H213	109.5	C45—C44—H44	120.2
C211—C212—H214	109.5	C43—C44—H44	120.2
C213—C212—H214	109.5	C46—C45—C44	120.45 (19)
H213—C212—H214	108.1	C46—C45—H45	119.8
C214—C213—C212	114.65 (18)	C44—C45—H45	119.8
C214—C213—H215	108.6	C45—C46—C47	120.27 (18)
C212—C213—H215	108.6	C45—C46—H46	119.9
C214—C213—H216	108.6	C47—C46—H46	119.9
C212—C213—H216	108.6	C46—C47—C42	119.8 (2)
H215—C213—H216	107.6	C46—C47—H47	120.1
C213—C214—H217	109.5	C42—C47—H47	120.1
C213—C214—H218	109.5	C412—C411—Sn4	113.43 (13)
H217—C214—H218	109.5	C412—C411—H411	108.9
C213—C214—H219	109.5	Sn4—C411—H411	108.9
H217—C214—H219	109.5	C412—C411—H412	108.9
H218—C214—H219	109.5	Sn4—C411—H412	108.9
C222—C221—Sn2	117.13 (13)	H411—C411—H412	107.7
C222—C221—H221	108.0	C413—C412—C411	112.25 (18)
Sn2—C221—H221	108.0	C413—C412—H413	109.2
C222—C221—H222	108.0	C411—C412—H413	109.2
Sn2—C221—H222	108.0	C413—C412—H414	109.2
H221—C221—H222	107.3	C411—C412—H414	109.2
C223—C222—C221	112.96 (18)	H413—C412—H414	107.9
C223—C222—H223	109.0	C414—C413—C412	110.6 (3)
C221—C222—H223	109.0	C414—C413—H415	109.5
C223—C222—H224	109.0	C412—C413—H415	109.5
C221—C222—H224	109.0	C414—C413—H416	109.5
H223—C222—H224	107.8	C412—C413—H416	109.5
C222—C223—C224	112.0 (2)	H415—C413—H416	108.1
C222—C223—H225	109.2	C413—C414—H417	109.5
C224—C223—H225	109.2	C413—C414—H418	109.5
C222—C223—H226	109.2	H417—C414—H418	109.5
C224—C223—H226	109.2	C413—C414—H419	109.5
H225—C223—H226	107.9	H417—C414—H419	109.5
C223—C224—H227	109.5	H418—C414—H419	109.5
C223—C224—H228	109.5	C416—C415—Sn4	111.5 (4)
H227—C224—H228	109.5	C416—C415—H431	109.3
C223—C224—H229	109.5	Sn4—C415—H431	109.3
H227—C224—H229	109.5	C416—C415—H432	109.3
H228—C224—H229	109.5	Sn4—C415—H432	109.3
C11—O11—Sn1	130.39 (12)	H431—C415—H432	108.0
C11—O12—Sn2	136.33 (12)	C417—C416—C415	108.1 (6)
O12—C11—O11	124.52 (16)	C417—C416—H433	110.1
O12—C11—C12	117.39 (16)	C415—C416—H433	110.1
O11—C11—C12	118.09 (16)	C417—C416—H434	110.1
C13—C12—C17	119.84 (18)	C415—C416—H434	110.1
C13—C12—C11	120.83 (18)	H433—C416—H434	108.4
C17—C12—C11	119.32 (17)	C418—C417—C416	118.3 (15)
C12—C13—C14	119.5 (2)	C418—C417—H435	107.7
C12—C13—H13	120.3	C416—C417—H435	107.7
C14—C13—H13	120.3	C418—C417—H436	107.7
C15—C14—C13	120.1 (2)	C416—C417—H436	107.7
C15—C14—H14	119.9	H435—C417—H436	107.1
C13—C14—H14	119.9	C417—C418—H437	109.5
C16—C15—C14	120.6 (2)	C417—C418—H438	109.5
C16—C15—H15	119.7	H437—C418—H438	109.5
C14—C15—H15	119.7	C417—C418—H439	109.5
C15—C16—C17	119.6 (2)	H437—C418—H439	109.5
C15—C16—H16	120.2	H438—C418—H439	109.5
C17—C16—H16	120.2	C422—C421—Sn4	119.24 (15)
C16—C17—C12	120.3 (2)	C422—C421—H421	107.5
C16—C17—H17	119.8	Sn4—C421—H421	107.5
C12—C17—H17	119.8	C422—C421—H422	107.5
C21—O21—Sn2	110.21 (10)	Sn4—C421—H422	107.5
O22—C21—O21	122.11 (16)	H421—C421—H422	107.0
O22—C21—C22	121.51 (15)	C423—C422—C421	117.2 (3)
O21—C21—C22	116.38 (15)	C423—C422—H423	108.0
C27—C22—C23	119.73 (17)	C421—C422—H423	108.0
C27—C22—C21	118.77 (17)	C423—C422—H424	108.0
C23—C22—C21	121.50 (16)	C421—C422—H424	108.0
C24—C23—C22	120.09 (18)	H423—C422—H424	107.3
C24—C23—H23	120.0	C424—C423—C422	114.8 (3)
C22—C23—H23	120.0	C424—C423—H425	108.6
C25—C24—C23	120.1 (2)	C422—C423—H425	108.6
C25—C24—H24	119.9	C424—C423—H426	108.6
C23—C24—H24	119.9	C422—C423—H426	108.6
C26—C25—C24	120.10 (18)	H425—C423—H426	107.6
C26—C25—H25	120.0	C423—C424—H427	109.5
C24—C25—H25	120.0	C423—C424—H428	109.5
C25—C26—C27	120.60 (19)	H427—C424—H428	109.5
C25—C26—H26	119.7	C423—C424—H429	109.5
C27—C26—H26	119.7	H427—C424—H429	109.5
C22—C27—C26	119.37 (19)	H428—C424—H429	109.5
C22—C27—H27	120.3	C426—C425—Sn4	118.6 (3)
C26—C27—H27	120.3	C426—C425—H441	107.7
O3—Sn3—C321	104.42 (6)	Sn4—C425—H441	107.7
O3—Sn3—C311	113.05 (6)	C426—C425—H442	107.7
C321—Sn3—C311	141.73 (7)	Sn4—C425—H442	107.7
O3—Sn3—O3^ii^	75.78 (5)	H441—C425—H442	107.1
C321—Sn3—O3^ii^	98.78 (7)	C425—C426—C427	109.7 (5)
C311—Sn3—O3^ii^	97.63 (6)	C425—C426—H443	109.7
O3—Sn3—O31	88.63 (5)	C427—C426—H443	109.7
C321—Sn3—O31	90.42 (7)	C425—C426—H444	109.7
C311—Sn3—O31	83.17 (6)	C427—C426—H444	109.7
O3^ii^—Sn3—O31	163.43 (5)	H443—C426—H444	108.2
O3—Sn4—C411	107.63 (6)	C428—C427—C426	114.1 (8)
O3—Sn4—O41	78.49 (5)	C428—C427—H445	108.7
C411—Sn4—O41	99.90 (7)	C426—C427—H445	108.7
O3—Sn4—O32	90.57 (5)	C428—C427—H446	108.7
C411—Sn4—O32	90.61 (7)	C426—C427—H446	108.7
O41—Sn4—O32	166.67 (5)	H445—C427—H446	107.6
Sn4—O3—Sn3	135.16 (6)	C427—C428—H447	109.5
Sn4—O3—Sn3^ii^	120.55 (5)	C427—C428—H448	109.5
Sn3—O3—Sn3^ii^	104.22 (5)	H447—C428—H448	109.5
C312—C311—Sn3	115.78 (12)	C427—C428—H449	109.5
C312—C311—H311	108.3	H447—C428—H449	109.5
Sn3—C311—H311	108.3	H448—C428—H449	109.5
			
C111—C112—C113—C114	−175.8 (2)	Sn1—C111—C112—C113	177.18 (15)
C121—C122—C123—C124	171.8 (2)	Sn1—C121—C122—C123	−172.39 (15)
C211—C212—C213—C214	−178.65 (19)	Sn2—C211—C212—C213	179.79 (14)
C221—C222—C223—C224	175.83 (19)	Sn2—C221—C222—C223	−177.13 (14)
C311—C312—C313—C314	−179.97 (19)	Sn3—C311—C312—C313	179.85 (13)
C321—C322—C323—C324	−173.66 (19)	Sn3—C321—C322—C323	167.95 (14)
C411—C412—C413—C414	−179.9 (4)	Sn4—C411—C412—C413	68.3 (2)
C415—C416—C417—C418	−177.5 (16)	Sn4—C415—C416—C417	−72.2 (7)
C421—C422—C423—C424	55.9 (5)	Sn4—C421—C422—C423	167.1 (3)
C425—C426—C427—C428	79.6 (11)	Sn4—C425—C426—C427	176.8 (5)

Symmetry codes: (i) −*x*, −*y*+2, −*z*; (ii) −*x*+1, −*y*+1, −*z*+1.
